# Primary total knee arthroplasty assisted by computed tomography-free navigation for secondary knee osteoarthritis following massive calcium phosphate cement packing for distal femoral giant-cell bone tumor treatment: a case report

**DOI:** 10.1186/s12891-022-05131-0

**Published:** 2022-02-22

**Authors:** Akihiko Takeuchi, Norio Yamamoto, Takaaki Ohmori, Katsuhiro Hayashi, Shinji Miwa, Kentaro Igarashi, Takashi Higuchi, Kensaku Abe, Hirotaka Yonezawa, Sei Morinaga, Yoshihiro Araki, Yohei Asano, Shiro Saito, Hiroyuki Tsuchiya

**Affiliations:** Department of Orthopaedic Surgery, Graduate School of Medical Sciences, Kanazawa University, 13-1 Takara-machi, Kanazawa-shi, Ishikawa-ken 920-8641 Japan

**Keywords:** GCTB, CPC, CT-free navigation, TKA

## Abstract

**Background:**

Giant cell tumor of bone (GCTB) is an intermediate tumor commonly arising from the epiphysis of the distal femur and proximal tibia. Standard GCTB treatment is joint-preserving surgery performed using thorough curettage and the filling of the cavity with allo-, auto-, polymethyl methacrylate (PMMA), or synthetic bone graft. Calcium phosphate cement (CPC) is an artificial bone substitute, which has the benefit of being able to adjust defects, consequently inducing immediate mechanical strength, and promoting biological healing. Secondary osteoarthritis may occur following GCTB treatment and may need additional surgery if severe. However, details regarding surgery for secondary osteoarthritis have not been fully elucidated. There are no reports on the use of total knee arthroplasty (TKA) for the treatment of secondary osteoarthritis following CPC packing. The insertion of an alignment rod is a standard procedure in TKA; however, it was difficult to perform in this case due to CPC. Therefore, we used a computed tomography (CT)-free navigation system to assist the distal femur cut. This study presents a knee joint secondary osteoarthritis case following CPC packing for GCTB curettage that was treated with standard TKA.

**Case presentation:**

A 67-year-old Japanese woman, who was previously diagnosed with left distal femur GCTB and was treated by curettage and CPC packing 7 years ago, complained of severe knee pain. Left knee joint plain radiography revealed Kellgren and Lawrence (K-L) grade 4 osteoarthritis without evidence of tumor recurrence. Therefore, she was scheduled for TKA. There are no reports on the cutting of a femoral condyle surface with massive CPC with accurate alignment. Because it is difficult to insert the alignment rod intramedullary and cut the femoral condyle with CPC, we planned CT-free navigation-guided surgery for accurate bone cutting using an oscillating tip saw system to prevent CPC cracks. We performed standard TKA without complications, as planned. Postoperative X-ray showed normal alignment. Knee Society Knee Score (KSKS) and Knee Society Function Score (KSFS) ameliorated from 27 and 29 to 64 and 68, respectively The patient can walk without a cane postoperatively.

**Conclusion:**

There was no report about the surface TKA guided by CT-free navigation after primary GCT surgery with CPC. We believe that this case report will help in planning salvage surgery for secondary osteoarthritis after CPC packing.

**Supplementary Information:**

The online version contains supplementary material available at 10.1186/s12891-022-05131-0.

## Background

Giant cell tumor of bone (GCTB) is an intermediate tumor typically arising from the epiphysis of the distal femur, proximal tibia, distal radius, and proximal humerus and occasionally arising from the metaphysis [1]. Standard GCTB treatment is joint-preserving surgery by thorough curettage with adjuvant treatment and the filling of the cavity with allo- [2], auto- [3], polymethyl methacrylate (PMMA) [4–6], or synthetic bone graft [7, 8], enabling joint preservation; the local recurrence rate was reported to be up to 30%. The grading system based on the radiological tumor extension of the Campanacci grading system has been widely used to predict the local recurrence [9]. Calcium phosphate cement (CPC) is an artificial bone substitute that has the benefit of being able to adjust defects with CPC molding, inducing immediate mechanical strength, preserving the bone stock, and promoting biological healing [8, 10]. GCTB is located close to the joint; therefore, secondary osteoarthritis is a notable complication [11] that requires additional surgery if severe [12–14]. However, to date, details regarding additional surgery for severe secondary osteoarthritis have not been fully elucidated. Only two cases of standard total knee arthroplasty (TKA) for secondary osteoarthritis treatment have been reported [15, 16]. Inserting an alignment rod is a standard procedure in TKA; however, it was difficult to perform in this case due to CPC. Therefore, we used a computed tomography (CT)-free navigation system [17, 18]. There was no published report about the surface TKA guided by CT-free navigation after primary GCT surgery with CPC.

## Case presentation

**Fig. 1:**
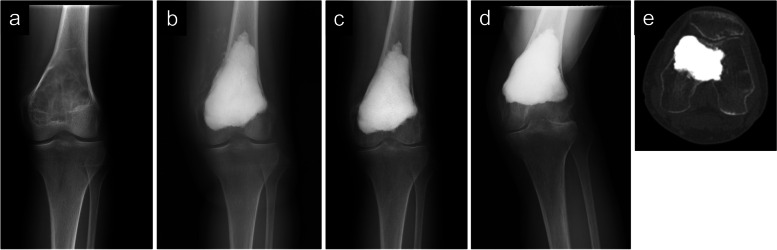
Preoperative radiogram images. Preoperative plain radiograms of the left knee joint showed an osteolytic lesion with scalloping (**a**). A postoperative plain radiogram showed that the cavity was filled with calcium phosphate cement (**b**). A plain radiogram after 5 years showed a K-L grade 3 joint space narrowing (**c**). A plain radiogram after 6 years showed the progressive joint space narrowing; the narrowing by the time of this radiogram was graded as K-L grade 4 (lateral tibiofemoral) and grade 2 (medial tibiofemoral) osteoarthritis (**d**). A CT imaging showed K-L grade 4 patellofemoral osteoarthritis

**Fig. 2:**
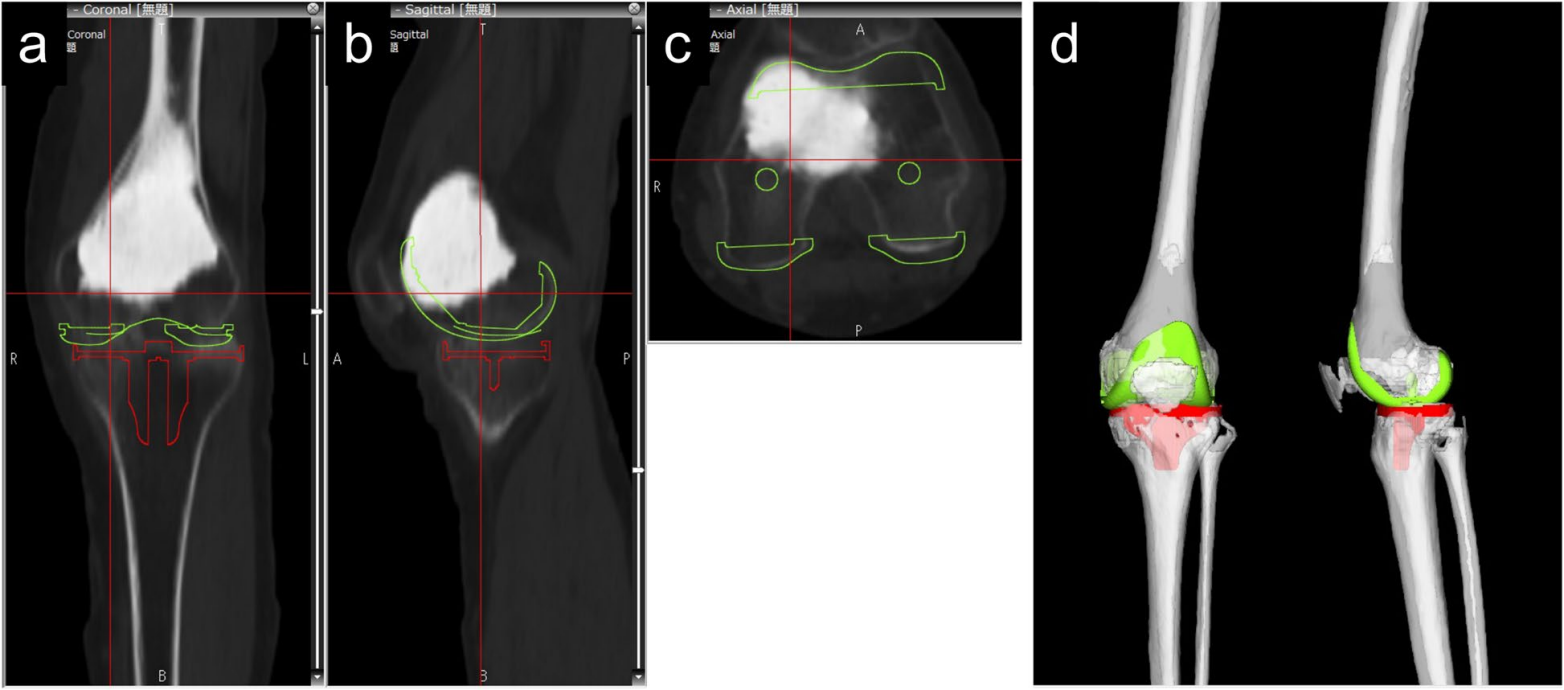
Three-dimensional templating imaging (**a**: coronal, **b**: sagittal, **c**: axial, **d**: 3D view) for computed tomography-free navigation assisted-surgery. The distal anterior part of calcium phosphate cement needed to be resected (**b, c**). The size and alignment of the component were three-dimensionally visualized (**d**)

**Fig. 3:**
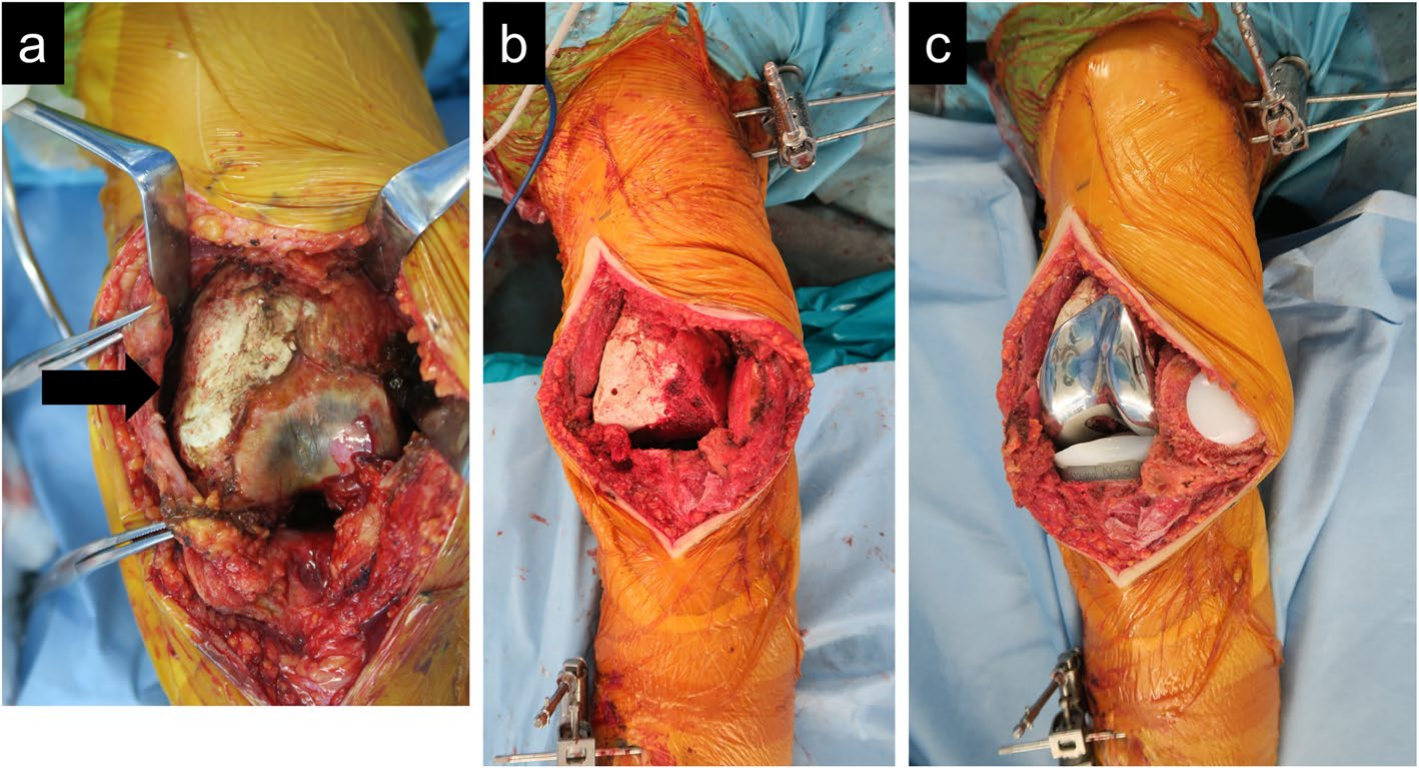
Intraoperative photographs. Degenerative changes in the femoral articular surface and synovitis were observed. The black arrow showed calcium phosphate cement (**a**). After femoral and tibial cutting, no crack was observed. Two trackers were set on the femur and tibia (**b**). The femoral and tibial components were placed. The patella was also resurfaced (**c**)

**Fig. 4:**
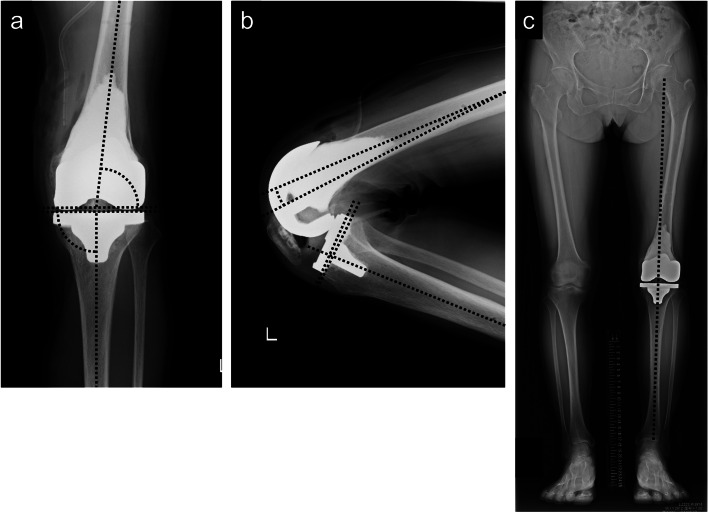
Postoperative radiogram images. Postoperative radiograms showed that the implant was placed with accurate alignment (anterior-posterior view [**a**]: aLDFA: 83.4° and MPTA: 89.8°; and lateral view [**b**]: femoral component flexion: 5.6° and tibial tilt: 3.8°). Whole leg radiograms in a standing position taken 1 year postoperatively showed that the mechanical axis passed the center of the tibial component (**c**). aLDFA: anatomical lateral distal femur angle, MPTA: medial proximal tibia angle

**Fig. 5:**
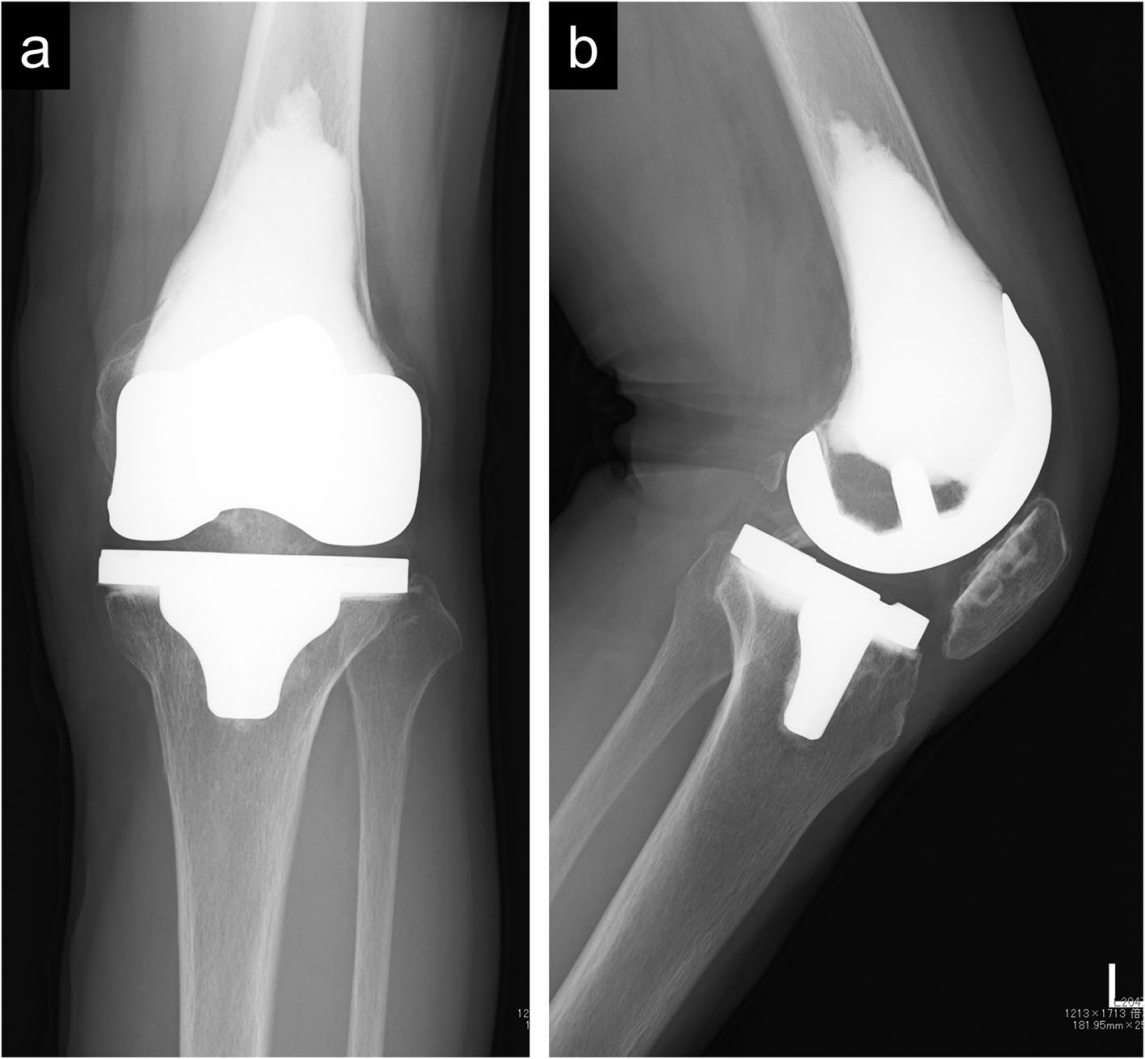
Plain radiogram images. Plain radiograms taken 4 years postoperatively showed no implant loosening. Anterior-posterior view (**a**). Lateral view (**b**)

A 67-year-old Japanese woman (body mass index [BMI], 16.9), who was initially diagnosed with left distal femur GCTB (Fig. 1a, sup. Fig. 1) and graded as Campanacci grade II, was treated with phenol and ethanol adjuvant curettage and the filling of the cavity with CPC (Biopex®-R; Pentax Co., Tokyo, Japan) following the intraoperative pathological diagnosis of GCTB (Fig. 1b). The final diagnosis by permanent section was also confirmed as GCTB (sup. Fig. 2a). The postoperative course was uneventful; however, 5 years postoperatively, she gradually started complaining of knee pain (Fig. 1c). The pain subsequently became severe, and magnetic resonance imaging 5 years postoperatively revealed synovitis without apparent local recurrence (sup. Fig. 3). Needle biopsy confirmed chronic synovitis (sup. Fig. 2b), and plain radiogram 6 years postoperatively showed K-L grade 4 (lateral tibiofemoral and patellofemoral) and grade 2 (medial tibiofemoral) osteoarthritis (Fig. 1d, e) [19, 20]. Moreover, the patient complained of a restricted knee range of motion (− 20 to 130 degrees); however, she did not show lateral instability. Knee Society Knee Score (KSKS) and Knee Society Function Score (KSFS) were 27 and 29, respectively [21]. Preoperative radiogram showed K-L grade 1 osteoarthritis that progressed during the last 6 years, without major risk factors, including high BMI (> 30) and injury, other than the female sex and older age (> 65 years old) [22, 23]. Therefore, we diagnosed the case as secondary osteoarthritis. Standard TKA was initially planned; however, cutting the femoral surface using an intramedullary alignment nail was seemingly difficult due to considerable amounts of CPC. Furthermore, we were concerned about the risk of CPC cracks during femoral surface cutting using a standard bone saw. Therefore, we planned to use a CT-free navigation system (Navigation: Stryker Navigation Cart System: Stryker Orthopaedics, Mahwah, NJ, USA, Software: Stryker Knee Navigation Ver2.0: Stryker Orthopaedics, Mahwah, NJ, USA), which enables accurate bone cutting without an intramedullary alignment rod. Preoperative planning was performed using a 3D-templating software (ZedView, ZedKnee; LEXI Co., Ltd., Tokyo, Japan) to determine the size and position of the implant (Fig. 2). Intraoperatively, the knee joint was approached via a midline skin incision, and the suprapatellar pouch was filled with the proliferative synovial tissue. CPC and degenerative joint surface were exposed (Fig. 3a) following synovial tissue resection, which was histologically diagnosed as chronic synovitis (sup. Fig. 2c); two truckers were set on the femur and tibia. According to a previous report, we set the mechanical axes of both the femur and tibia as an anatomical index for the image-free navigation system [24]. First, in the femur, we registered at the center of the femoral head, lateral and medial epicondyles of the femur, surface of the medial and lateral condyle, Whiteside line, and knee center. Next, in the tibia, we registered at the anterior-posterior axis, lateral and medial malleolus of the ankle, medial and lateral tibial articular surface, and knee center. After registration, cutting blocks were set at the distal end of the femur and proximal end of the tibia perpendicular to their bone axes. The amount of femoral osteotomy was defined as the thickness of the implant from the femur medial joint surface, and the amount of tibial osteotomy was determined using the fibula head height. A total of 9 mm of the distal medial condyle was cut using abductor rotation, as determined by the surgical epicondylar axis (SEA). The anterior and posterior surfaces were cut via a cutting block using a Stryker Precision® blade and a Stryker Precision System 6 saw (Stryker Instruments) according to the anterior surface, which enabled the cutting of CPC without cracks. Subsequently, the tibial cutting guide was mounted using the support of navigation (Fig. 3b). After the tibial surface was cut, the implant trial was placed, and the flexion and extension gap were assessed. Finally, the component (Triathlon CR [Stryker]) was implanted with cement; 4-, 3-, and 9-mm inserts were used for the femur, tibia, and CS, respectively (Fig. 3c). The patella was resurfacing. A postoperative radiogram showed the precise alignment of the component (Fig. 4a, b) and mechanical axis, which was cited at the center of the knee joint (Fig. 4c). The postoperative course was uneventful without any adverse event. Four years later, radiogram showed good alignment and no loosening of the component (Fig. 5). The knee range of motion was 0 to 95 degrees. KSKS and KSFS ameliorated to 64 and 68, respectively.

## Discussion and conclusions

This case demonstrates that standard TKA combined with CT-free navigation assistance can treat knee joint secondary osteoarthritis following CPC packing for GCTB treatment. CPC packing in the initial surgery was performed to preserve the bone stock with the biological interface and long-lasting stability; therefore, the authors planned to keep the bone stock without CPC damage. The challenge, in this case, was that the large amount of CPC packed in the distal part of the femur prevents the insertion of the intramedullary rod. To accurately cut the femoral condyle, we used a CT-guided navigation system. Moreover, a standard bone saw has the risk of causing CPC cracks [25, 26]. Therefore, we used an oscillating tip saw system [27] instead and successfully cut the surface without cracks.

In this case, although GCTB was close to the joint, curettage and the filling of the cavity using biological or artificial substitutes are standard procedures. PMMA is frequently used to fill the large defect following curettage [4–6]. The mechanical strength of PMMA was reported to be > 70 megapascals (mPa) [28] whereas that of CPC was 30–40 mPa [29]. Compared with PMMA, CPC has the properties of a biological interface [7] and is reported to strengthen the torsional stress of an animal long bone after filling [25]. Recent advancements in denosumab (a receptor activator of nuclear factor-κB ligand inhibitor) could induce tumor shrinkage, providing a chance to perform joint-preserving surgery instead of joint replacement [30]. Conversely, joint-preserving surgery may induce secondary osteoarthritis, with an incidence of 1.7–33% [11, 12, 14, 31]; however, details regarding salvage surgery have not been fully elucidated, suggesting that information on secondary osteoarthritis treatment is also unclear. Conti et al. reported patellofemoral joint arthroplasty as the salvage surgery for femoropatellar osteoarthritis following the initial treatment of curettage and PMMA packing for distal femoral GCTB. Monocompartmental or bicompartmental knee replacement could be applicable, depending on the degree of osteoarthritis; however, Conti et al. recommended total knee replacement as a stable and definitive alternative in older adult patients who have limited activities [32]. The patient, in this case, presented with K-L grade 4 (lateral tibiofemoral and patellofemoral) and grade 2 (medial tibiofemoral) osteoarthritis and was an older adult; therefore, total knee arthroplasty was performed. There are only two reports on the use of standard TKA, which preserves both lateral ligaments, to treat secondary osteoarthritis [15, 16]. Zylberberg et al. reported successful secondary osteoarthritis replacement using standard TKA and reported that the cutting of the surface and the insertion of a nail did not influence PMMA existence [15]. However, implant alignment accuracy was not fully mentioned. Lyall et al. reported the salvage of secondary osteoarthritis following an autograft using a stem augmented femoral component [16]. Different from the abovementioned two cases, the cavity after curettage, in this case, was filled with massive CPC, showing good integration with the surrounding bone. However, due to its nature, the artificial substitute was not remodeled entirely and had a risk of cracking by rough cutting or nailing. We easily suspected that there will be difficulty in the insertion of the intramedullary rod and a risk of CPC cracking by the standard bone saw or nailing. Therefore, we planned to use a CT-free navigation system that enabled the mounting of the cutting block without nail insertion. The technology of the navigation system allowed accurate bone cutting alignment, ligament balancing, and the evaluation of the symmetry of the flexion and extension gaps [17]. Several reports have confirmed that navigation-assisted TKA is more reliable in achieving precise alignment than conventional TKA using intramedullary/extramedullary alignment techniques [33–35]. We confirmed that navigation-assisted surgery was useful to perform primary TKA for secondary osteoarthritis following GCTB curettage and CPC packing. Preoperative CT-based planning was also useful for planning bone cutting and determining the implant size [36]. This case had distal femoral scalloping due to tumor extension; therefore, the filled CPC expanded anteriorly, inducing a risk of registration error. We also used the oscillating tip saw system for gentle cutting. Peters et al. reported that the oscillating tip saw system demonstrated reduced noise emission compared with the conventional saw system [37]. We considered that the low noise indicated low vibration, consequently reducing CPC crack risk. A patient-specific guide [38] or robotic surgery [39] may be promising, less invasive, and more concise.

In conclusion, we treated a case of secondary knee osteoarthritis following primary GCTB surgery with CPC using TKA assisted by a CT-free navigation system, which was not previously reported. We believe that this case report will help in planning salvage surgery for secondary osteoarthritis after CPC packing. Navigation-guided TKA leaves the bone stock intact and may offer the best result compared with other surgeries.

## Supplementary Information

**Additional file 1: Sup. Fig. 1.** Preoperative magnetic resonance imaging revealed the tumor expanding anteriorly, with mixed low- and iso-intensity in T1-weighted image (WI) (a, d), and mixed iso- and high- intensity in short inversion time inversion recovery (STIR) T1-WI (b) and T2-WI (e). The gadolinium-enhanced image showed tumor enhancement (c, f). Coronal view (a, b, c). Axial view (d, e, f).

**Additional file 2: Sup. Fig. 2.** Histological analysis of the primary tumor revealed mononuclear, multinucleated cells and foam cells, which were compatible with giant cell tumor of bone (a). Biopsy specimen presented the proliferation of synovial lining cells with inflammatory cells (b). Excised synovial tissue specimen showed the proliferation of synovial lining cells, inflammatory cells, and hemosiderin deposition (hematoxylin and eosin stain; scale bar, 100 μm).

**Additional file 3: Sup. Fig. 3.** Magnetic resonance imaging after 5 years showed no tumor recurrence (coronal view: a. T1-WI, b. STIR T1-WI, c. STIR T2-WI), and joint fluid retention and synovial lesion in the suprapatellar pouch (axial view: d. T1-WI, b. STIR T1-WI, c. STIR T2-WI).

## Data Availability

All data generated or analyzed during this study are included in this published article.

## Abbreviations

GCTB: Giant cell tumor of bone
PMMA: Polymethyl methacrylate
CPC: Calcium phosphate cement
CT: Computed tomography
K-L: Kellgren and Lawrence
KSKS: Knee Society Knee Score
KSFS: Knee Society Function Score
TKA: Total knee arthroplasty
SEA: Surgical epicondylar axis
AP view: Anteroposterior view
aLDFA: Anatomical lateral distal femur angle
MPTA: Medial proximal tibia angle
WI: Weighted image
STIR: Short inversion time inversion recovery

## References

[1] Flanagan A, Larousserie F, O’Donnell P, Yoshida A, The WHO Classification of Tumours Editorial board (2020). Giant cell tumours of bone. WHO Classification of Tumours Soft Tissue and Bone Tumours.

[2] Ayerza MA, Aponte-Tinao LA, Farfalli GL, Restrepo CAL, Muscolo DL (2009). Joint preservation after extensive curettage of knee giant cell tumors. Clin Orthop Relat Res.

[3] Blackley HRL, Davis AM, Hutchison CR, Gross AE (2001). Proximal femoral allografts for reconstruction of bone stock in revision arthroplasty of the hip. A nine to fifteen-year follow-up. J Bone Joint Surg Am.

[4] Fraquet N, Faizon G, Rosset P, Phillipeau JM, Waast D, Gouin F (2009). Long bones giant cells tumors: treatment by curretage and cavity filling cementation. Orthop Traumatol Surg Res.

[5] Bini SA, Gill K, Johnston JO. Giant cell tumor of bone. Curettage and cement reconstruction. Clin Orthop Relat Res. 1995;321:245–50. 7497676

[6] Turcotte RE, Wunder JS, Isler MH, Bell RS, Schachar N, Masri BA (2002). Giant cell tumor of long bone: a Canadian sarcoma group study. Clin Orthop Relat Res.

[7] Matsumine A, Kusuzaki K, Matsubara T, Okamuka A, Okuyama N, Miyazaki S (2006). Calcium phosphate cement in musculoskeletal tumor surgery. J Surg Oncol.

[8] Takeuchi A, Suwanpramote P, Yamamoto N, Shirai T, Hayashi K, Kimura H (2018). Mid- to long-term clinical outcome of giant cell tumor of bone treated with calcium phosphate cement following thorough curettage and phenolization. J Surg Oncol.

[9] Campanacci M, Baldini N, Boriani S, Sudanese A (1987). Giant-cell tumor of bone. J Bone Joint Surg Am.

[10] Higuchi T, Yamamoto N, Hayashi K, Takeuchi A, Kimura H, Miwa S (2018). Calcium phosphate cement in the surgical management of benign bone tumors. Anticancer Res.

[11] Suzuki Y, Nishida Y, Yamada Y, Tsukushi S, Sugiura H, Nakashima H (2007). Re-operation results in osteoarthritic change of knee joints in patients with giant cell tumor of bone. Knee..

[12] van der Heijden L, van de Sande MAJ, Heineken AC, Fiocco M, Nelissen RGHH, Dijkstra PDS (2013). Mid-term outcome after curettage with polymethylmethacrylate for giant cell tumor around the knee: higher risk of radiographic osteoarthritis?. J Bone Joint Surg Am.

[13] Caubère A, Harrosch S, Fioravanti M, Curvale G, Rochwerger A, Mattei JC (2017). Does curettage–cement packing for treating giant cell tumors at the knee lead to osteoarthritis?. Orthop Traumatol Surg Res.

[14] Araki Y, Yamamoto N, Hayashi K, Takeuchi A, Miwa S, Igarashi K (2020). Secondary osteoarthritis after curettage and calcium phosphate cementing for Giant-cell tumor of bone around the knee joint. JBJS Open Access.

[15] Zylberberg A, Bayley G, Gala L, Kim PR (2015). Primary Total knee Arthroplasty twenty years after distal femoral cement augmentation of a Giant cell tumor. Case Rep Orthop.

[16] Lyall H, El-Zebdeh M, Ireland J (2009). Primary total knee arthroplasty performed 20 years after treatment for giant cell tumor. J Knee Surg.

[17] Jones CW, Jerabek SA (2018). Current role of computer navigation in Total knee Arthroplasty. J Arthroplast.

[18] Yamamura K, Minoda Y, Mizokawa S, Ohta Y, Sugama R, Nakamura S (2017). Novel alignment measurement technique for total knee arthroplasty using patient specific instrumentation. Arch Orthop Trauma Surg.

[19] Kellgren JH, Lawrence JS (1957). Radiological assessment of Osteo-arthrosis. Ann Rheum Dis.

[20] Stoddart JC, Dandridge O, Garner A, Cobb J, van Arkel RJ (2021). The compartmental distribution of knee osteoarthritis – a systematic review and meta-analysis. Osteoarthr Cartil.

[21] Scuderi GR, Bourne RB, Noble PC, Benjamin JB, Lonner JH, Scott WN (2012). The new knee society knee scoring system. Clin Orthop Relat Res.

[22] Toivanen AT, Heliövaara M, Impivaara O, Arokoski JPA, Knekt P, Lauren H (2010). Obesity, physically demanding work and traumatic knee injury are major risk factors for knee osteoarthritis-a population-based study with a follow-up of 22 years. Rheumatology..

[23] Zhang Y, Jordan JM (2010). Epidemiology of osteoarthritis. Clin Geriatr Med.

[24] Ohmori T, Maeda T, Kabata T, Kajino Y, Iwai S, Tsuchiya H (2015). The accuracy of initial bone cutting in Total knee Arthroplasty. Open J Orthop.

[25] Mizobuchi H, Tani T, Takemasa R, Yamamoto H, Sonobe H (2002). Mechanical properties of the femur filled with calcium phosphate cement under torsional loading: a model in rabbits. J Orthop Sci.

[26] Naito K, Obayashi O, Mogami A, Itoi A, Kaneko K (2008). Fracture of the calcium phosphate bone cement which used to enchondroma of the hand: a case report. Eur J Orthop Surg Traumatol.

[27] Feczko PZ, Fokkenrood HJP, van Assen T, Deckers P, Emans PJ, Arts JJ (2017). Accuracy of the precision saw versus the sagittal saw during total knee arthroplasty: a randomised clinical trial. Knee..

[28] Lee C (2005). The mechanical properties of PMMA bone cement. Well-cemented Total hip Arthroplast Theory Pract.

[29] Unuma H, Matsushima Y (2013). Preparation of calcium phosphate cement with an improved setting behavior. J Asian Ceram Soc.

[30] Rutkowski P, Ferrari S, Grimer RJ, Stalley PD, Dijkstra SPD, Pienkowski A (2015). Surgical downstaging in an open-label phase II trial of denosumab in patients with giant cell tumor of bone. Ann Surg Oncol.

[31] Blackley HR, Wunder JS, Davis AM, White LM, Kandel R, Bell RS (1999). Treatment of giant-cell tumors of long bones with curettage and bone-grafting. J Bone Joint Surg Am.

[32] Conti A, Boffano M, Pellegrino P, Ratto N, Sabatini L, Piana R (2020). Femoropatellar osteoarthritis and trochlear femoral bone defect due to giant cell tumor of the knee: a selected patellofemoral joint arthroplasty and reconstructive technique a case report. JBJS Case Connect.

[33] Bäthis H, Perlick L, Tingart M, Lüring C, Zurakowski D, Grifka J (2004). Alignment in total knee arthroplasty. A comparison of computer-assisted surgery with the conventional technique. J Bone Joint Surg Br.

[34] Anderson KC, Buehler KC, Markel DC (2005). Computer assisted navigation in Total knee Arthroplasty. J Arthroplast.

[35] Keyes BJ, Markel DC, Meneghini RM (2013). Evaluation of limb alignment, component positioning, and function in primary total knee arthroplasty using a pinless navigation technique compared with conventional methods. J Knee Surg.

[36] Pietrzak JRT, Rowan FE, Kayani B, Donaldson MJ, Huq SS, Haddad FS (2019). Preoperative CT-based three-dimensional Templating in robot-assisted Total knee Arthroplasty more accurately predicts implant sizes than two-dimensional Templating. J Knee Surg.

[37] Peters MP, Feczko PZ, Tsang K, van Rietbergen B, Arts JJ, Emans PJ (2016). Noise exposure in TKA surgery; oscillating tip saw systems vs oscillating blade saw systems. J Arthroplast.

[38] Gaukel S, Vuille-Dit-Bille RN, Schläppi M, Koch PP. CT-based patient-specific instrumentation for total knee arthroplasty in over 700 cases: single-use instruments are as accurate as standard instruments. Knee Surg Sports Traumatol Arthrosc. 2020. 10.1007/s00167-020-06150-x. Epub ahead of print. 10.1007/s00167-020-06150-x PMC8866287 32676744

[39] Ofa SA, Ross BJ, Flick TR, Patel AH, Sherman WF (2020). Robotic Total knee Arthroplasty vs conventional Total knee Arthroplasty: a Nationwide database study. Arthroplast Today.

