# Supplementary figures and images for: Primary total knee arthroplasty assisted by computed tomography-free navigation for secondary knee osteoarthritis following massive calcium phosphate cement packing for distal femoral giant-cell bone tumor treatment: a case report
Source: BMC Musculoskelet Disord. 2022 Feb 22;23:170. doi: 10.1186/s12891-022-05131-0 (PMC8864852; doi:10.1186/s12891-022-05131-0)

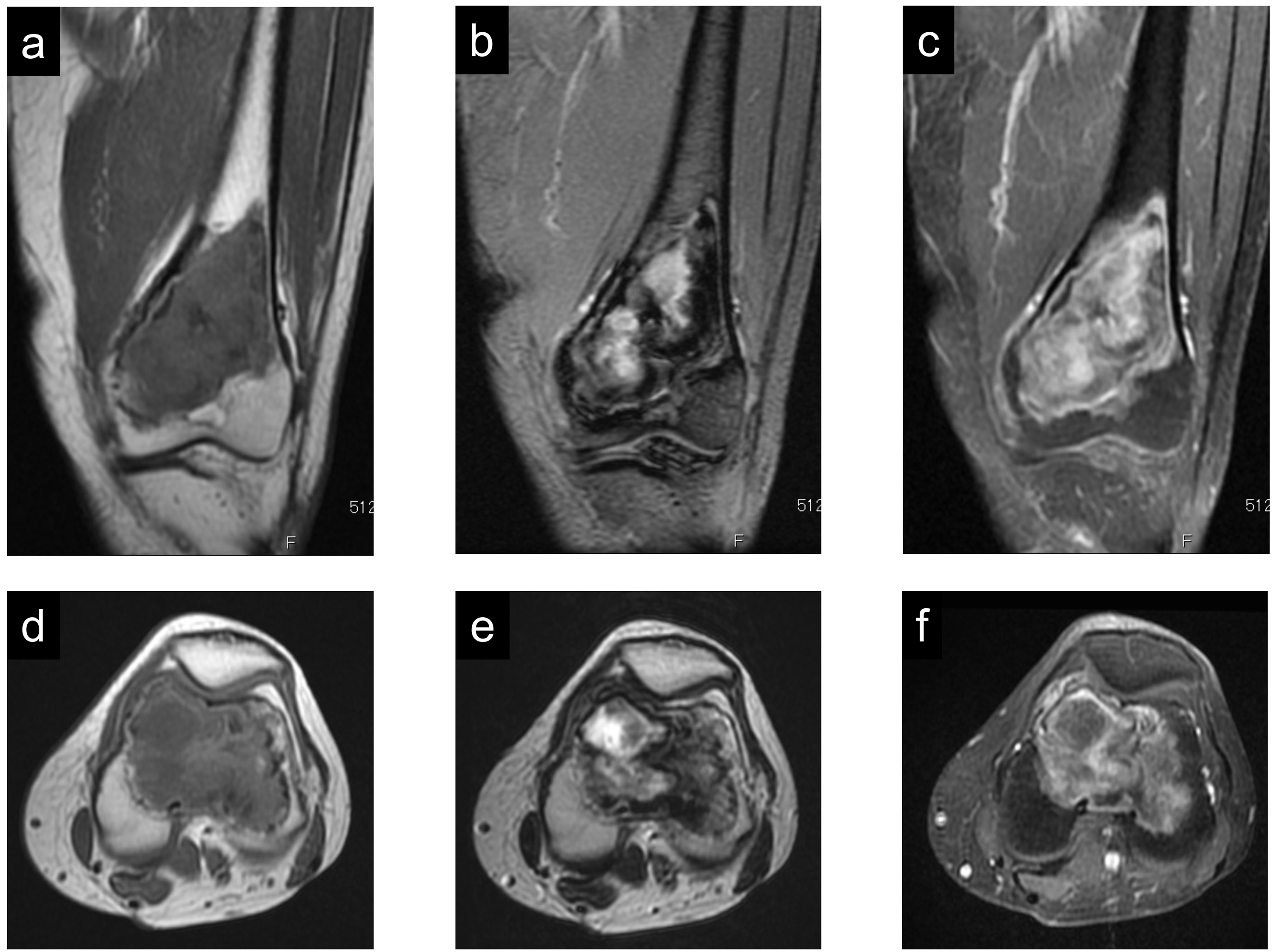

Supplement: Supplementary file 1 — Additional file 1: Sup. Fig. 1. Preoperative magnetic resonance imaging revealed the tumor expanding anteriorly, with mixed low- and iso-intensity in T1-weighted image (WI) (a, d), and mixed iso- and high- intensity in short inversion time inversion recovery (STIR) T1-WI (b) and T2-WI (e). The gadolinium-enhanced image showed tumor enhancement (c, f). Coronal view (a, b, c). Axial view (d, e, f). [file 12891_2022_5131_MOESM1_ESM.tif]

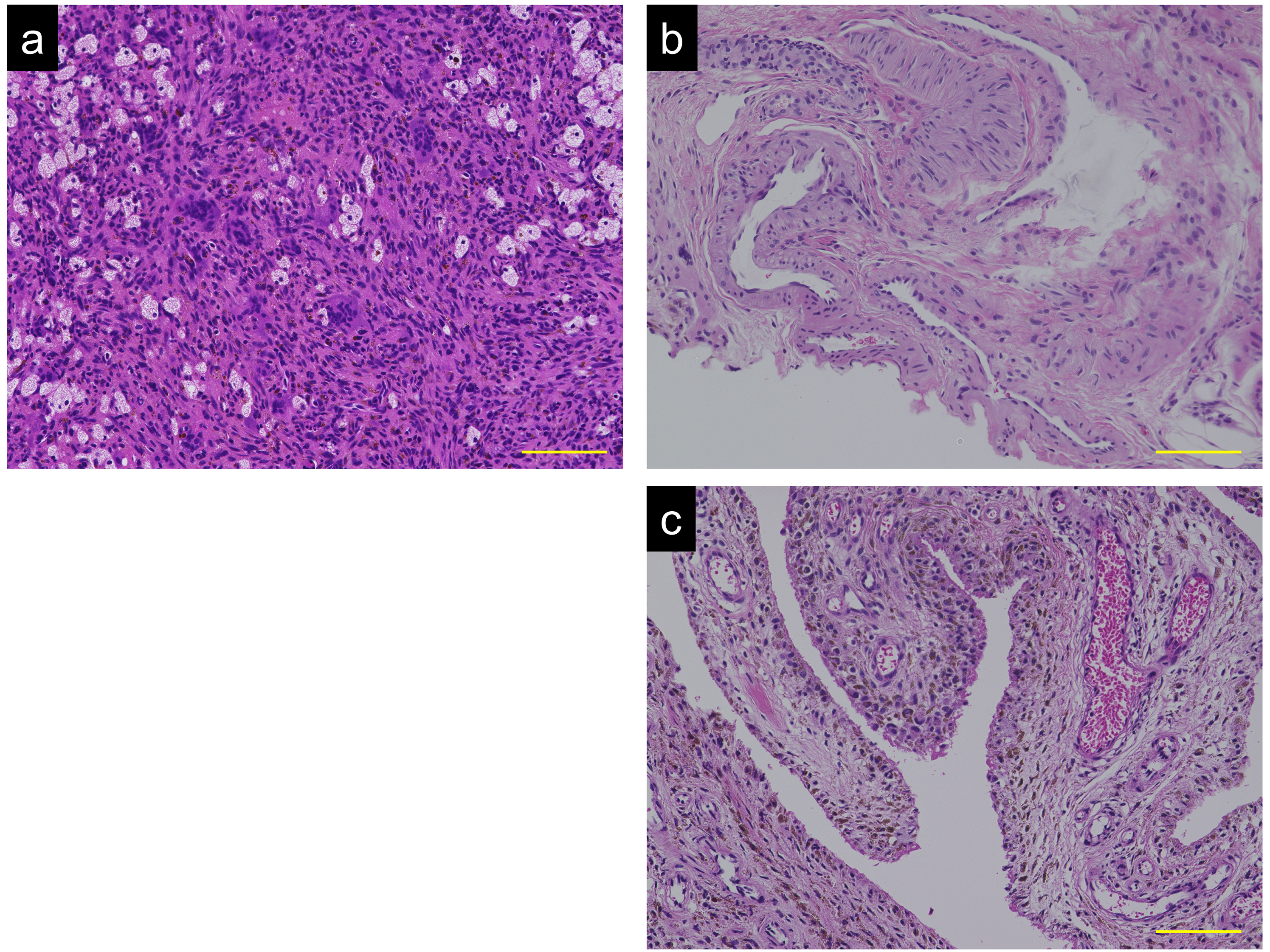

Supplement: Supplementary file 2 — Additional file 2: Sup. Fig. 2. Histological analysis of the primary tumor revealed mononuclear, multinucleated cells and foam cells, which were compatible with giant cell tumor of bone (a). Biopsy specimen presented the proliferation of synovial lining cells with inflammatory cells (b). Excised synovial tissue specimen showed the proliferation of synovial lining cells, inflammatory cells, and hemosiderin deposition (hematoxylin and eosin stain; scale bar, 100 μm). [file 12891_2022_5131_MOESM2_ESM.tif]

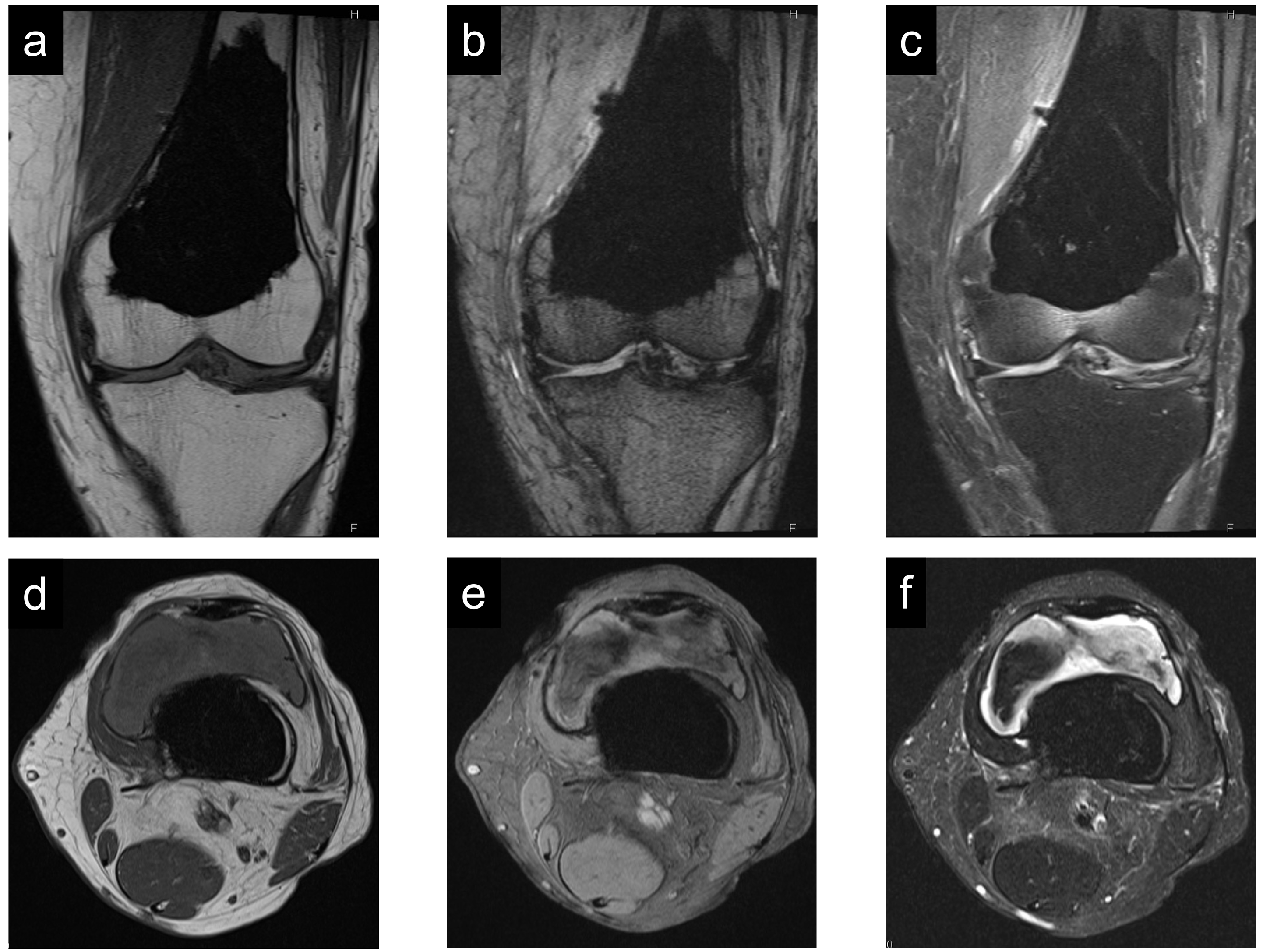

Supplement: Supplementary file 3 — Additional file 3: Sup. Fig. 3. Magnetic resonance imaging after 5 years showed no tumor recurrence (coronal view: a. T1-WI, b. STIR T1-WI, c. STIR T2-WI), and joint fluid retention and synovial lesion in the suprapatellar pouch (axial view: d. T1-WI, b. STIR T1-WI, c. STIR T2-WI). [file 12891_2022_5131_MOESM3_ESM.tif]
